# Ascorbate sensitizes human osteosarcoma cells to the cytostatic effects of cisplatin

**DOI:** 10.1002/prp2.632

**Published:** 2020-07-29

**Authors:** Naohiro Oka, Akiyoshi Komuro, Hisayuki Amano, Suman Dash, Masahiko Honda, Kazushige Ota, Shunji Nishimura, Takeshi Ueda, Masao Akagi, Hitoshi Okada

**Affiliations:** ^1^ Department of Orthopedics Faculty of Medicine Kindai University Osaka Japan; ^2^ Graduate School of Medical Sciences Faculty of Medicine Kindai University Osaka Japan; ^3^ Department of Biochemistry Faculty of Medicine Kindai University Osaka Japan; ^4^ Anti‐aging Center Kindai University Osaka Japan

**Keywords:** chemoresistance, metabolism, mitochondria, osteosarcoma, ROS

## Abstract

Osteosarcoma (OS) is the most common malignant bone tumor and a leading cause of cancer‐related deaths in children and adolescents. Current standard treatments for OS are a combination of preoperative chemotherapy, surgical resection, and adjuvant chemotherapy. Cisplatin is used as the standard chemotherapeutic for OS treatment, but it induces various adverse effects, limiting its clinical application. Improving treatment efficacy without increasing the cisplatin dosage is desirable. In the present study, we assessed the combined effect of ascorbate on cisplatin treatment using cultured human OS cells. Co‐treatment with ascorbate induced greater suppression of OS cell but not nonmalignant cell proliferation. The chemosensitizing effect of ascorbate on cisplatin treatment was tightly linked to ROS production. Altered cellular redox state due to increased ROS production modified glycolysis and mitochondrial function in OS cells. In addition, OS cell sphere formation was markedly decreased, suggesting that ascorbate increased the treatment efficacy of cisplatin against stem‐like cells in the cancer cell population. We also found that enhanced MYC signaling, ribosomal biogenesis, glycolysis, and mitochondrial respiration are key signatures in OS cells with cisplatin resistance. Furthermore, cisplatin resistance was reversed by ascorbate. Taken together, our findings provide a rationale for combining cisplatin with ascorbate in therapeutic strategies against OS.

**Abbreviations**
2‐DG: 2‐deoxy‐D‐glucose
AA: antimycin A
CSC: cancer stem cell
DHE: dihydroethidium
ECAR: extracellular acidification rate
FCCP: carbonyl cyanide‐p‐trifluoromethoxy phenylhydrazone
G6PD: glucose‐6‐phosphate dehydrogenase
HK2: hexokinase 2
LDHA: lactate dehydrogenase A
MCT4: monocarboxylate transporter 4
OCR: oxygen consumption rate
OS: osteosarcoma
PPP: pentose phosphate pathway
ROS: reactive oxygen species
ROT: rotenone

**Significance Statement**

Chemoresistance is a major cause of cancer mortality. We show that co‐treatment with cisplatin and ascorbate induces higher ROS production and metabolic shift in osteosarcoma (OS) cells, leading to strong suppression of cell proliferation. In addition, acquired cisplatin resistance in OS cells is reversed by co‐treatment with ascorbate, rationalizing a combination of cisplatin and ascorbate for OS treatment.

## 1 INTRODUCTION

Osteosarcoma (OS) is the most common primary malignant bone tumor and affects adolescents and children.^1^ In addition, OS is the most frequent cause of cancer‐related deaths in children and adolescents.^2^ The current standard treatment for OS is a combination of chemotherapy and surgical resection.^3^ The overall survival for patients with localized OS is approximately 65%‐70%.^4^ In recent decades, improved chemotherapy regimens have contributed to treatment outcomes,^5^ but more than 30% of patients have recurrence and metastasis.^6^ The response to preoperative chemotherapy is an important predictive factor for the prognosis of OS.^7^ Thus, several attempts have been made to improve the treatment efficacy of poor responders using modified chemotherapy protocols, but a unified strategy has not been agreed upon.^5^ The use of high‐dose chemotherapeutic drugs for primary chemotherapy could be a solution, but systemic toxicity ^8^ and a risk of secondary cancer are concerns.^9^, ^10^ Thus, there is a pressing need to develop new strategies or modify current chemotherapy regimens to treat OS patients refractory to chemotherapy.

Cisplatin is one of the most widely used platinum‐based anticancer drugs for treating a variety of solid tumors, including OS.^11^ Cisplatin interacts with various cellular components, including chromosomal DNA, proteins, small peptides, lipids, and RNA,^12^, ^13^, ^14^, ^15^ resulting in suppression of tumor cell proliferation via multiple pathways.^15^ In addition, cisplatin treatment induces oxidative stress, which contributes to its cytotoxic effects.^16^, ^17^ The adverse effects of cisplatin place limits on its clinical application.^18^, ^19^ Thus, the development of strategies to improve cisplatin treatment efficacy without increasing dose is important.

The anticancer effects of ascorbate (vitamin C) were proposed in the 1950s,^20^, ^21^ but contradictory results were reported in subsequent studies.^22^ When administered intravenously at high doses, ascorbate has exhibited clinical and preclinical potential in cancer treatment, especially in synergy with other chemotherapeutic agents.^23^, ^24^ Additional studies have shown that ascorbate has an antitumor effect in a number of cancer models, including pancreatic, ovarian, and breast.^25^, ^26^, ^27^, ^28^, ^29^ Several molecular mechanisms have been proposed for the cytotoxic effects of ascorbate, including increased pro‐oxidant damage by generation of reactive oxygen species (ROS), but little is known about the effect of ascorbate on the cisplatin response in human OS. Normally, ROS are produced mainly by intracellular aerobic respiration and metabolism, consequently influencing cell and tissue homeostasis. An altered redox balance due to increasing ROS, however, is known to have pathophysiological effects, including oxidative damage of surrounding lipids, proteins, and DNA. It is thus important to better understand the effects of combined cisplatin and ascorbate treatment on the oxidative stress response in OS, including consequential effects on mitochondrial function and metabolic shift.

In the present study, we show that ascorbate enhances the antitumor effect of cisplatin via increased ROS production, and that the treatment of OS cells with cisplatin and ascorbate alters glycolysis and mitochondrial function. In addition, the co‐treatment of OS cells with cisplatin and ascorbate reduces sphere formation, suggesting a chemosensitizing activity of ascorbate on cancer cells that retain cancer stem cell (CSC) properties.

## 2 MATERIALS AND METHODS

### 2.1 Cell lines and cell culture

Human OS cell lines, U2OS and 143B, were obtained from Cell Bank, RIKEN BioResource Research Center (Tsukuba, Ibaraki, Japan). A nonmalignant human lung fibroblast line, IMR‐90 (ATCC CCL‐186), was obtained from the American Type Culture Collection (ATCC). U2OS cells were maintained in McCoy's 5A medium (Thermo Fisher Scientific), 143B cells in Dulbecco's Modified Eagle Medium (Thermo Fisher Scientific), and IMR‐90 cells in Eagle's Minimum Essential Medium (Thermo Fisher Scientific), cultured at 37°C in a humidified atmosphere containing 5% CO_2_. All cell culture media were supplemented with 10% heat‐inactivated fetal bovine serum (FBS; JRH, Nichirei Biosciences, Tokyo, Japan), penicillin (100 units/ml; Thermo Fisher Scientific), and streptomycin (100 µg/mL; Thermo Fisher Scientific). Cisplatin was obtained from FUJIFILM Wako Pure Chemical Corporation. N‐acetyl‐cysteine (NAC) and ascorbate were purchased from Sigma‐Aldrich (Merck Millipore). Two cisplatin‐resistant U2OS cell lines were established as described previously for non‐small cell lung cancer ^30^ with a modification of cisplatin dose. Briefly, 1 × 10^6^ U2OS cells were cultured on three 10‐cm dishes in the presence or absence of cisplatin. The cisplatin concentration was increased from 1 to 30 μmol/L over 6 months. The medium was changed every other day with occasional passage to maintain appropriate cellular confluency. Cisplatin resistance was confirmed by a cell proliferation assay.

### 2.2 Cell viability assay

Cellular viability was assessed using Cell Count Reagent SF (WST‐8) (Nacalai tesque). U2OS, 143B, and IMR‐90 cells were seeded on 96‐well plates (1500 cells/well), 12 hours before starting treatment with cisplatin (1 nmol/L, 10 nmol/L, 100 nmol/L, 1 μmol/L, 10 μmol/L, and 30 μmol/L), ascorbate (1 nmol/L, 10 nmol/L, 100 nmol/L, 1 μmol/L, and 10 μmol/L), or cisplatin plus ascorbate. Ninety‐six hours after treatment, 10 µL of WST‐8 (2‐(2‐methoxy‐4‐nitrophenyl)‐3‐(4‐nitrophenyl)‐5‐(2,4‐disulfophenyl)‐2H‐tetrazolium) was added to each well and incubated for 2 hours at 37°C, after which the absorbance at 450 nm was measured immediately on a microplate reader (iMark; Bio‐Rad). The background readings were subtracted from each original reading. The cell viability assay was performed in triplicate and repeated at least three times. The IC_50_ was calculated from the curves constructed by plotting cellular viability vs drug concentration. A drug combination effect between cisplatin and ascorbate was evaluated by Combination Index.^31^

### 2.3 Measurement of intracellular ROS

Intracellular ROS levels were measured by dihydroethidium (DHE) staining. U2OS cells were treated with cisplatin, ascorbate, or cisplatin plus ascorbate, or left untreated for 96 hours. The cells were trypsinized, collected in McCoy's 5A medium supplemented with 10% FBS, centrifuged at 1200 rpm at 4°C for 5 minutes, and then resuspended and incubated in phosphate‐buffered saline (PBS) supplemented with 10 μmol/L DHE and 1% FBS at 37°C for 30 minutes in dark. After washing with PBS/1% FBS, cells were resuspended in 200‐µL PBS/1% FBS and analyzed by flow cytometry (Canto II; BD Biosciences) and FlowJo (Becton Dickinson and Company). Intracellular ROS levels were determined by measuring the mean fluorescence intensity (MFI) of DHE‐positive cells. The MFI of the treated cells was expressed relative to MFI of the untreated cells. ROS measurements were performed in triplicate and the entire experiment was repeated at least three times.

### 2.4 Live‐cell metabolic assay

Cellular metabolism and mitochondrial function were measured as the extracellular acidification rate (ECAR) and oxygen consumption rate (OCR) using a Seahorse Bioscience XFe96 Extracellular Flux Analyzer (Agilent Technologies). U2OS cells were seeded in the wells (1 × 10^4^ cells/well) of a standard XFe96 microplate (Agilent Technologies) and incubated at 37°C. Twelve hours later, the cells were treated with cisplatin, ascorbate, or cisplatin plus ascorbate, or left untreated for 96 hours and analyzed using the flux analyzer. Analysis medium supplemented with 1‐mmol/L pyruvic acid, 10‐mmol/L glucose, and 2‐mmol/L glutamine (pH 7.4) was freshly prepared according to the manufacturer's protocol. For the measurement of mitochondrial and glycolytic functions, the assay conditions, including the final concentrations of FCCP (1.0 µmol/L), oligomycin (1.0 µmol/L), rotenone plus antimycin A (ROT/AA; 0.5 µmol/L), and 2‐deoxy‐D‐glucose (2‐DG; 50 mmol/L), were chosen based on our preliminary experiments. We routinely confirmed no significant changes in the number of cells in each well. The flux analyzer measurements were performed in triplicate and repeated at least three times.

### 2.5 Gamma‐H2AX immunofluorescence, imaging, and image analysis

U2OS cells were seeded at a density of 1 × 10^4^ cells/well on a sterile round cover glass (Matsunami) set into a well of a 12‐well plate. Cells were treated with cisplatin, ascorbate, or cisplatin plus ascorbate, or left untreated for 96 hours and then fixed with 4% paraformaldehyde in phosphate buffer solution (FUJIFILM Wako) for 5 minutes at room temperature. Fixed cells were permeabilized with Tris‐buffered saline (TBS)/0.1% Triton X‐100 at room temperature for 5 minutes. After 1 hour of blocking with TBS containing 2% bovine serum albumin at 37°C, the cells were incubated with mouse anti‐γH2AX (pS139) antibody (1/10 dilution; BD Biosciences) at 4°C overnight. After washing with TBS/0.1% Triton X‐100 three times, the cells were incubated with Alexa 488‐conjugated anti‐mouse antibody (1/200 dilution, Life Technologies) at room temperature for 1 hour. After washing again with TBS/Triton X‐100 three times, cover glasses were mounted on glass slides using an antifade mounting medium (Thermo Fisher Scientific) containing DAPI to counterstain DNA. The slides were imaged under a fluorescence microscope (BZ‐X710; Keyence, Osaka, Japan). For quantitative analysis, the number, size, and intensity of γH2AX foci in the nuclei were measured by image analysis software (Keyence). At least 10 views were randomly chosen from each slide for the analysis. γH2AX staining experiments were performed in triplicate and repeated three times.

### 2.6 Sphere formation assay

For the sphere formation assay, U2OS cells were resuspended in 0.33% agar gel and plated onto the top of a 0.66% agar gel layer in a 6‐well plate. McCoy's 5A medium containing cisplatin, ascorbate, or cisplatin plus ascorbate was added to cover the agar gel layers. Ninety‐six hours later, the medium containing the regents was removed and changed to normal McCoy's 5A medium. The medium was changed every other day and the number of spheres quantified 21 days posttreatment with cisplatin, ascorbate, or cisplatin plus ascorbate using inverted phase contrast microscopy (BZ‐X710, Keyence). At least 15 fields in each well were randomly picked and the size and number of spheres were measured. The sphere formation assays were performed in triplicate and repeated six times.

### 2.7 Real‐time RT‐PCR

Total RNA was extracted from U2OS cells using the Monarch Total RNA Miniprep kit (New England Biolabs) and reverse‐transcribed using the iScript^TM^ cDNA Synthesis Kit (Bio‐Rad). cDNAs were subjected to quantitative real‐time PCR using Luna Universal qPCR Master Mix (New England Biolabs). qPCR and data collection were performed using a StepOnePlus Real‐time PCR system (Thermo Fisher Scientific). The following primer pairs were used: human HK2, 5ʹ‐TGGAGATGGAGAATCAGA‐3ʹ/5ʹ‐CCAGGAAACTCTCGTCTA‐3ʹ; G6PD (glucose‐6‐phosphate dehydrogenase), 5ʹ‐CCGGATCGACCACTACCTGGGCAAG‐3ʹ /5ʹ‐GTTCCCCACGTACTGGCCCAGGACCA‐3ʹ; LDHA (lactate dehydrogenase A), 5ʹ‐GGTTGAGAGTGCTTATGA‐3ʹ/5ʹ‐AACACTAAGGAAGACATCA‐3ʹ; MCT4 (monocarboxylate transporter 4), 5ʹ‐ATTGGCCTGGTGCTGCTGATG‐3ʹ/5ʹ‐ CGAGTCTGCAGGAGGCTTGTG‐3ʹ; and HRPT1 (hypoxanthine phosphoribosyltransferase 1), 5ʹ‐TTTGCTTTCCTTGGTCA‐3ʹ /5ʹ‐GCTTGCGACCTTGACCATCT‐3ʹ.

### 2.8 RNA sequence analysis

RNA was extracted from cisplatin‐resistant and parental U2OS cells using an RNeasy Micro Plus kit (Qiagen) according to the manufacturer's protocol. RNA integrity was assessed on a Bioanalyzer (Agilent), and high‐quality samples were used for library preparation. RNAs were purified using the NEBNext Poly(A) mRNA Magnetic Isolation Module (New England Biolabs) and oligo‐dT beads, and the libraries were prepared using the SMARTer RNA‐seq kit (Takara Bio USA Inc). After normalization and pooling, the libraries were sequenced on a Nextseq 500 (Illumina) using 50 bp paired‐end reads to a depth of >30 million reads per sample. The quality of the RNA‐seq results was assessed using FastQC (ver. 0.11.7). The raw reads were trimmed and quality‐filtered using Trim Galore! (ver. 0.4.4), Trimmomatic (ver. 0.36),^32^ and cutadapt (ver. 1.16) software. Clean reads were aligned using STAR (ver. 2.6.1a),^33^ the count matrix generated using the featureCounts tool (ver. 1.6.1),^34^ and differential expression analysis performed using DESeq (ver. 1.30.0) ^35^ with standard settings. Genes with |log_2_FC| ≥ 2 and a *P*‐value < .05 were subjected to Gene Ontology analysis using clusterProfiler (ver. 3.6.0).^36^ We compared the gene expression levels from parental and cisplatin‐resistant U2OS cells and picked the genes with significant expression in gene set enrichment analysis (GSEA).^37^ The RNA sequence data have been deposited in the DDBJ database (accession number: DRA009407).

### 2.9 Statistical analysis

Differences between groups were analyzed by an unpaired two‐tailed Student's *t* test. Multiple groups were analyzed by one‐way analysis of variance. Results are presented as the mean ± standard deviation. *P* < .05 was considered significant.

## 3 RESULTS

### 3.1 Ascorbate enhances the cytotoxicity of cisplatin in human OS cells

**FIGURE 1:**
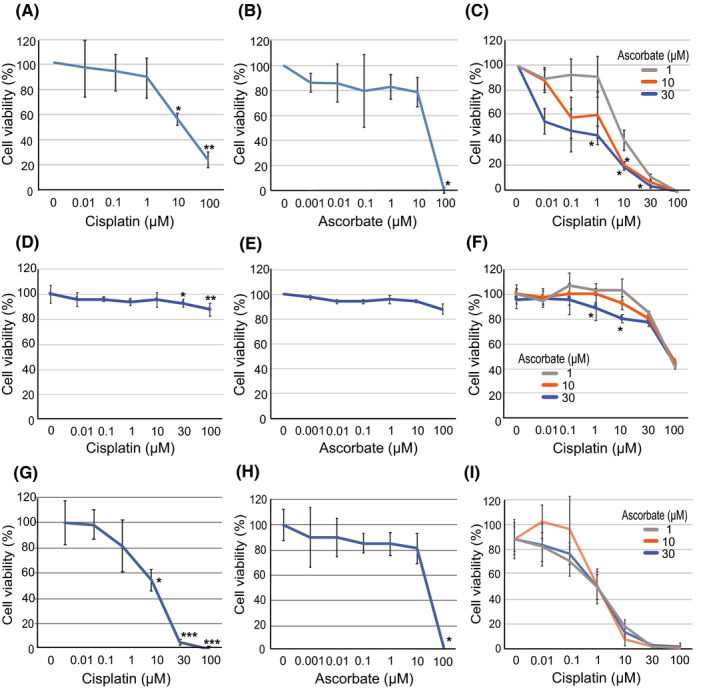
Ascorbate enhances the effect of cisplatin in osteosarcoma cells. A‐C, U2OS cells (1500 cells) were treated with cisplatin (0‐100 µmol/L) (A), ascorbate (0‐100 µmol/L) (B), and cisplatin (0‐100 µmol/L) plus ascorbate (1, 10, and 30 µmol/L) (C) for 96 h. D‐F, 143B cells (1,500 cells) were treated with cisplatin (0‐100 µmol/L) (D), ascorbate (0‐100 µmol/L) (E), and cisplatin (0‐100 µmol/L) plus ascorbate (1, 10, and 30 µmol/L) (F) for 96 h. G‐I, Nonmalignant human lung fibroblast, IMR‐90 cells (1,500 cells), were treated with cisplatin (0‐100 µmol/L) (G), ascorbate (0‐100 µmol/L) (H), and cisplatin (0‐100 µmol/L) plus ascorbate (1, 10, and 30 µmol/L) (I) for 96 h. Cell viability was quantified by the cell viability assay. The data represent the mean ± SD of triplicate samples from three independent experiments. *P < .05; **P < .01

To assess the effect of ascorbate on cisplatin‐induced cytotoxicity, we measured cellular viability after 96 hours of continuous cisplatin, ascorbate, or cisplatin plus ascorbate treatment. Cisplatin treatment decreased the viability of U2OS cells in a dose‐dependent manner with an IC_50_ of 15.5 μmol/L (Figure 1A). In contrast, ascorbate treatment alone did not significantly affect the viability of U2OS cells at doses between 0.001 and 10 μmol/L. At 100 μmol/L, ascorbate treatment markedly reduced cellular viability (Figure 1B). We next tested the chemosensitizing effect of ascorbate (1‐30 μmol/L) on cisplatin. Although ascorbate treatment alone did not affect cellular viability at these doses, it enhanced the cytotoxic effect of cisplatin (Figure 1C). The IC_50_ values for cisplatin upon combined treatment with cisplatin and ascorbate were 6.62 μmol/L with 1‐μmol/L ascorbate, 1.90 μmol/L with 10‐μmol/L ascorbate, and 0.06 μmol/L with 30‐μmol/L ascorbate. The Combination Index was 0.47 with 1‐μmol/L ascorbate and 0.56 with 10‐μmol/L ascorbate, showing the synergistic effect of the combined treatment. In 143B cells, cisplatin treatment slightly decreased cell viability, with an IC_50_ value of 532 μmol/L. The chemosensitizing effect of ascorbate on cisplatin was also observed in 143B cells. The IC_50_ values for combined treatment with ascorbate were 90.5 μmol/L with 1‐μmol/L ascorbate, 88.0 μmol/L with 10‐μmol/L ascorbate, and 80.7 μmol/L with 30‐μmol/L ascorbate. In contrast, ascorbate treatment did not affect the sensitivity of nonmalignant human lung fibroblast, IMR‐90 cells to cisplatin (Figure 1G). These data indicate that ascorbate treatment synergistically enhanced the cytotoxic effect of cisplatin in a dose‐dependent manner in human OS cells.

### 3.2 Synergistic ROS induction and DNA damage upon combined treatment with cisplatin and ascorbate

**FIGURE 2:**
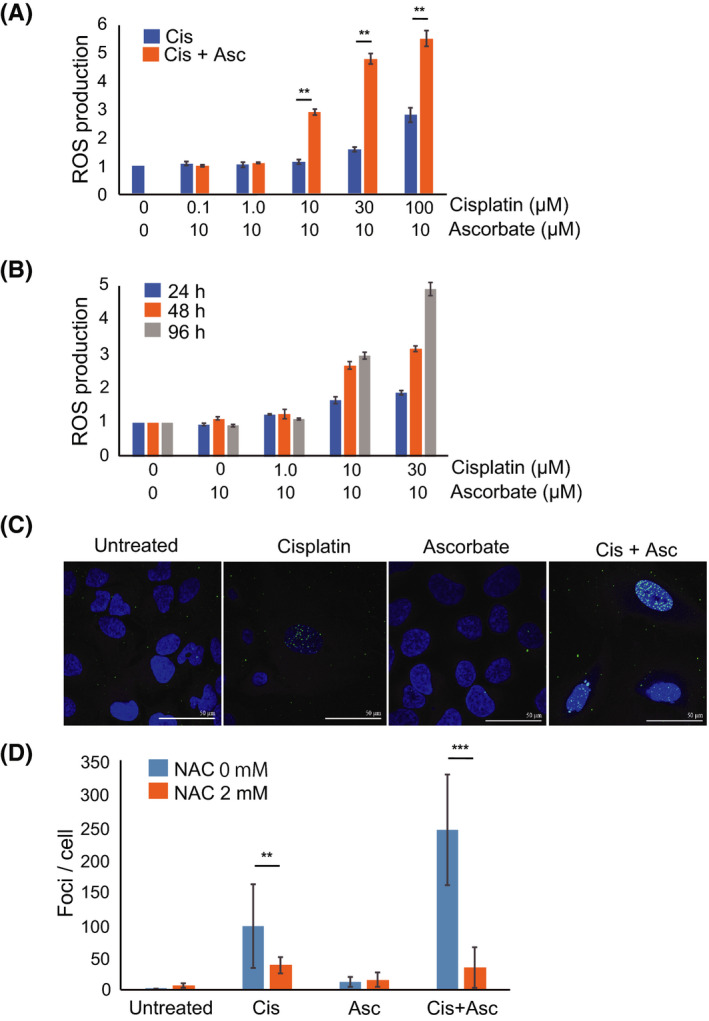
Ascorbate enhances ROS production in osteosarcoma cells. A, ROS levels in U2OS cells treated with cisplatin (0‐100 µmol/L) and ascorbate (10 µmol/L) for 96 h as measured by flow cytometry. Intracellular ROS levels were determined by measuring the mean fluorescence intensity (MFI) of DHE‐positive cells. MFI in the treated cells was expressed relative to MFI of the untreated cells (set at 1). B, ROS levels in U2OS cells measured by flow cytometry, 24, 48, and 96 h after treatment with cisplatin (0‐30 µmol/L) and ascorbate (10 µmol/L). MFI in the treated cells was expressed relative to MFI of the untreated cells (set at 1). C, U2OS cells were treated with cisplatin (10 µmol/L) and/or ascorbate (10 µmol/L) in the presence or absence of ROS scavenger NAC (2 mmol/L), 96 h after treatment and the number of γH2AX dots was counted. D, Immunostaining of γH2AX (green) and DAPI (blue). The data represent the mean ± SD of triplicate samples from three independent experiments. *P < .05; **P < .01

To gain insight into the potential mechanisms underlying the chemosensitizing effect of ascorbate on cisplatin treatment, we measured ROS production by DHE‐based flow cytometry. U2OS cells were continuously exposed to cisplatin or cisplatin plus ascorbate at the indicated doses for 96 hours and intracellular ROS levels were measured. Cisplatin treatment increased intracellular ROS levels in a dose‐dependent manner (Figure 2A). In addition, ROS levels significantly increased in the cells treated with cisplatin plus ascorbate compared to cisplatin treatment alone. To evaluate the kinetics of intracellular ROS production in response to treatment with cisplatin and ascorbate, we measured ROS levels after 24‐, 48‐, and 96‐hour exposure. Although ascorbate treatment alone did not increase intracellular ROS levels, the combined treatment results in an increase after 24 hours exposure, with further increase over time (Figure 2B). Hence, cisplatin and ascorbate together enhance intracellular ROS production in U2OS cells.

To evaluate the biological effect of the cisplatin‐induced increase in ROS, we examined double‐strand breaks in U2OS cells treated with cisplatin or cisplatin plus ascorbate by immunostaining for phosphorylated histone H2AX (γH2AX), which accumulates at the sites of double‐strand DNA damage. After 96 hours treatment of U2OS cells with cisplatin, ascorbate, or cisplatin plus ascorbate, we measured the number of γH2AX staining dots per cell. The number of γH2AX foci was comparable between untreated control cells and ascorbate‐treated cells, but significantly higher in cisplatin‐treated cells (Figure 2C,D). Combined treatment with cisplatin and ascorbate further increased the number of γH2AX foci than cisplatin treatment alone (Figure 2C,D). Less than 10% of cisplatin has been reported to bind DNA,^38^ but it is possible that the increase in γH2AX foci is due to this direct effect, enhanced by ascorbate. Thus, to confirm that ROS production significantly contributes to the observed DNA damage, we examined the effects of a ROS scavenger, NAC, on γH2AX foci formation. The addition of NAC markedly suppressed γH2AX foci formation (Figure 2D). We can conclude, therefore, that cisplatin‐induced ROS production leads to an increase in DNA damage, and that this effect is exacerbated upon addition of ascorbate.

### 3.3 Enhanced mitochondrial respiration altered the metabolism of U2OS cells

**FIGURE 3:**
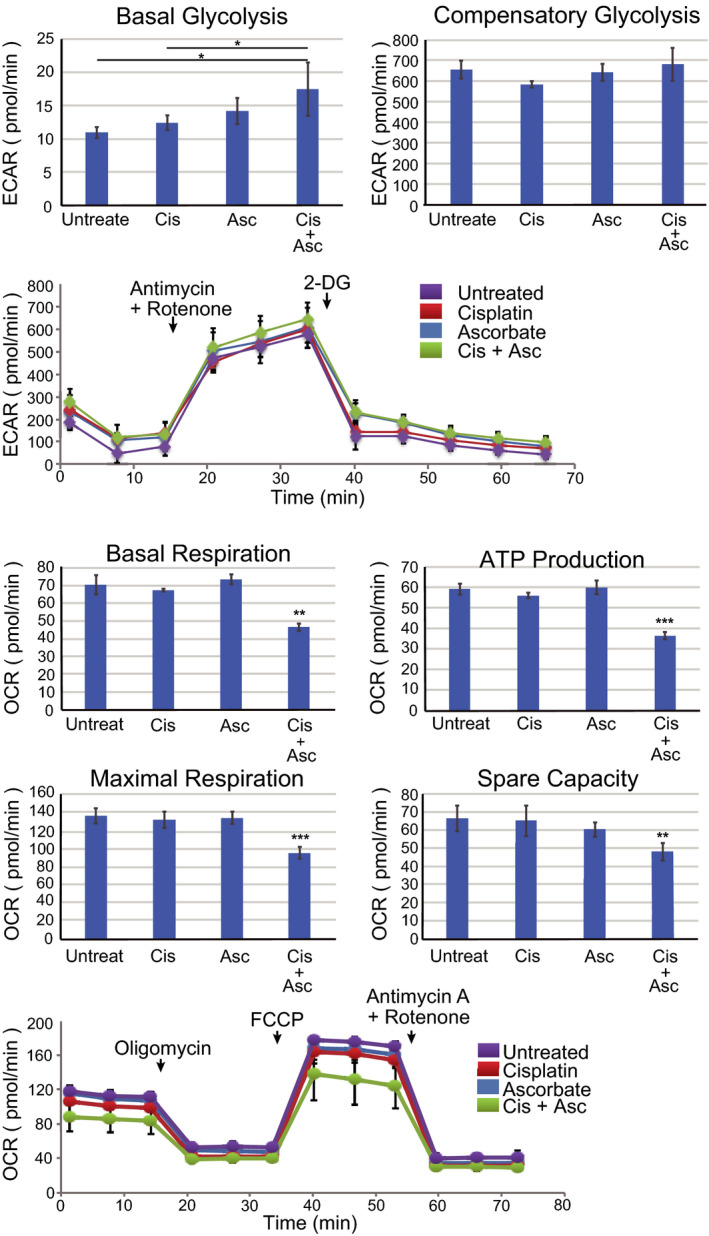
Ascorbate and cisplatin treatment alter mitochondrial respiration and glucose metabolism of U2OS cells. A, U2OS cells (1 × 10^4^ cells) were treated with cisplatin (10 µmol/L), ascorbate (10 µmol/L), or cisplatin plus ascorbate for 96 h and the glycolytic functions (basal and compensatory glycolysis) were measured by a flux analyzer. Kinetics of the ECAR response of U2OS cells to ROT/AA (0.5 µmol/L) or 2‐DG (50 mmol/L) was measured by a flux analyzer. B, U2OS cells (1 × 10^4^ cells) were treated with cisplatin (10 µmol/L), ascorbate (10 µmol/L), or both for 96 h and the mitochondrial functions (basal respiration, ATP production, maximal respiration, and spare capacity) were measured by a flux analyzer. Kinetics of the OCR response of U2OS cells to oligomycin (1.0 µmol/L), FCCP (1.0 µmol/L), or ROT/AA (0.5 µmol/L) was measured by a flux analyzer. The data represent the mean ± SD of triplicate samples from three independent experiments. *P < .05; **P < .01; ***P <.001

An altered cellular redox state due to increased ROS can shift the balance of metabolic processes in the cell. In particular, ROS can mediate pathophysiological effects via modulation of the glycolytic and mitochondrial respiratory pathways. Having established that cisplatin and cisplatin with ascorbate increase ROS production in OS cells, we thus examined the effect of these chemicals on glycolysis and mitochondrial function. Basal glycolysis was significantly increased by the combined treatment compared to no treatment or cisplatin treatment alone (Figure 3A). Compensatory glycolysis (assayed in the presence of mitochondrial inhibitors) was unchanged among U2OS cells treated with cisplatin, ascorbate, or cisplatin plus ascorbate, with a slight but insignificant increase observed in the cisplatin plus ascorbate‐treated cells (Figure 3A). In contrast to changes observed for basal glycolysis, mitochondrial function was decreased upon combined treatment with cisplatin and ascorbate compared to cisplatin treatment alone and no treatment (Figure 3B). U2OS cells treated with both chemicals exhibited lower basal respiration, ATP production, and levels of maximal respiration. The data indicate that cisplatin and ascorbate, together, increase ROS to levels that induce mitochondrial damage. A reduction in oxidative respiration is countered by elevated glycolytic flux in the treated U2OS cells, presumably as a circumventive measure.

### 3.4 Cisplatin and ascorbate treatment influences gene expression in the glycolytic and pentose phosphate pathways

**FIGURE 4:**
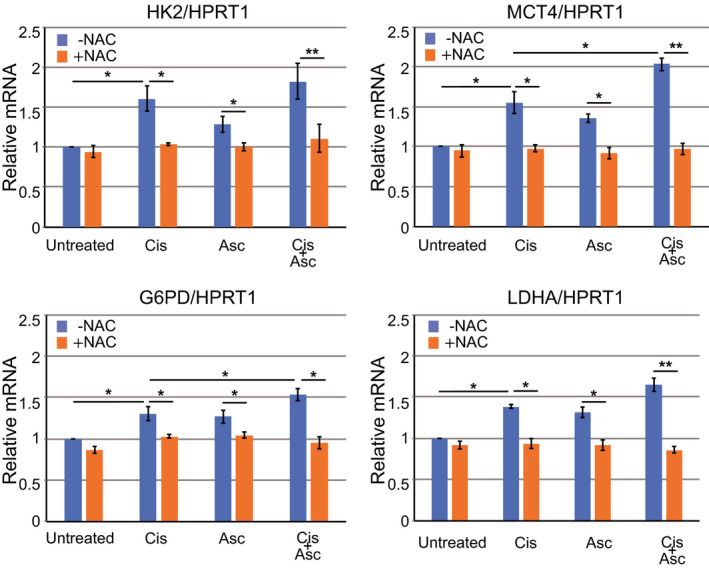
Cisplatin and ascorbate treatment affect the expression of rate‐limiting enzymes in glucose metabolism. qRT‐PCR analysis of the expression of hexokinase 2 (HK2), monocarboxylate transporter 4 (MCT4), glucose‐6‐phosphate dehydrogenase (G6PD), and lactate dehydrogenase A (LDHA) in U2OS cells treated with cisplatin (10 µmol/L), ascorbate (10 µmol/L), or both for 96 h in the presence or absence of NAC (2 mmol/L). HRPT1, control. Data are presented as the mean ± SD of triplicate samples from three independent experiments. *P < .05; **P < .01

Since combined treatment with cisplatin and ascorbate increases basal glycolysis, we sought to confirm and explain this increase by examining the expression of several genes involved in glucose metabolism. Firstly, we examined the levels of mRNA for HK2, the first enzyme in the glycolytic pathway, which phosphorylates glucose to produce glucose‐6‐phosphate. Cisplatin treatment increases HK2 mRNA levels (Figure 4). Subsequently, we examined MCT4 (required for extracellular excretion of lactic acid produced by glycolysis), LDHA (converts pyruvate to lactic acid in the glycolytic pathway), and G6PD. G6PD is the first enzyme in the PPP, a pathway which provides NADPH for the synthesis of fatty acids, steroids, and ribose (for nucleotide and nucleic acid formation) and for maintaining reduced glutathione (an antioxidant). MCT4 and G6PD were upregulated in U2OS cells treated by cisplatin, and the expression levels were further increased by the combined treatment with cisplatin plus ascorbate. LDHA was upregulated in U2OS cells treated with cisplatin but not significantly enhanced by the combined treatment (Figure 4).

Since increased ROS are known to affect metabolic pathways, we examined whether the enhanced gene expression observed here on treatment with cisplatin (and/or combined cisplatin and ascorbate) is linked directly to ROS production. RNA levels for the genes were, therefore, assessed in the presence of NAC, a ROS scavenger. NAC treatment significantly suppressed the induction of HK2, MCT4, LDHA, and G6PD (Figure 4). These data further support our findings that cisplatin and ascorbate synergistically modify mitochondrial function and glycolytic metabolism in U2OS cells, and that this is achieved, at least in part, by increased ROS production.

### 3.5 Combined treatment with cisplatin and ascorbate suppresses U2OS sphere formation

**FIGURE 5:**
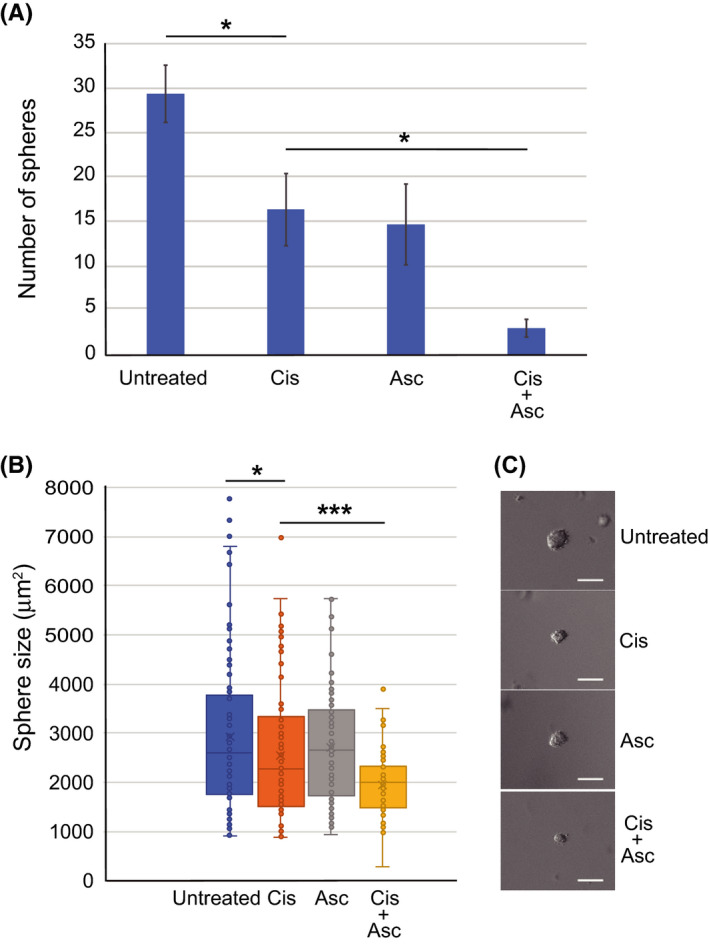
Cisplatin and ascorbate treatment reduced sphere formation. A, The difference in the number of spheres per microscopic field at 400× magnification. A representative result is shown (n = 15). B, The size distribution of spheres developed in cells treated with cisplatin, ascorbate, or cisplatin plus ascorbate (n = 70). C, Representative images of the spheres are shown (scale bar; 100 µm). The data represent the mean ± SD from three independent experiments. *P < .05; **P < .01; ***P < .001

A small subset of stem‐like cells present in tumors, known as CSCs, are proposed to be responsible for cancer initiation, and tumor recurrence, metastasis, and chemoresistance.^39^ Here, the sphere formation assay was used to identify a cell population with CSC properties in OS.^40^ The assay is based on the ability of CSCs to form a three‐dimensional sphere when grown in a gel matrix. To test whether combined treatment with cisplatin and ascorbate affects the sphere formation capacity of U2OS cells, the cells enclosed in agar gel were treated with cisplatin, ascorbate, or cisplatin plus ascorbate for 96 hours. The medium was changed to complete normal medium, and the culture continued for 21 days. Cisplatin plus ascorbate treatment significantly reduced the number and the average size of spheres, compared to untreated control and cisplatin or ascorbate treatment alone, or to untreated control (Figure 5A‐C). These findings suggest that cisplatin‐induced cytotoxicity is enhanced in the cells with sphere‐forming ability (CSCs) in the presence of ascorbate.

### 3.6 Ascorbate increases the chemosensitivity of cisplatin‐resistant U2OS cells

**FIGURE 6:**
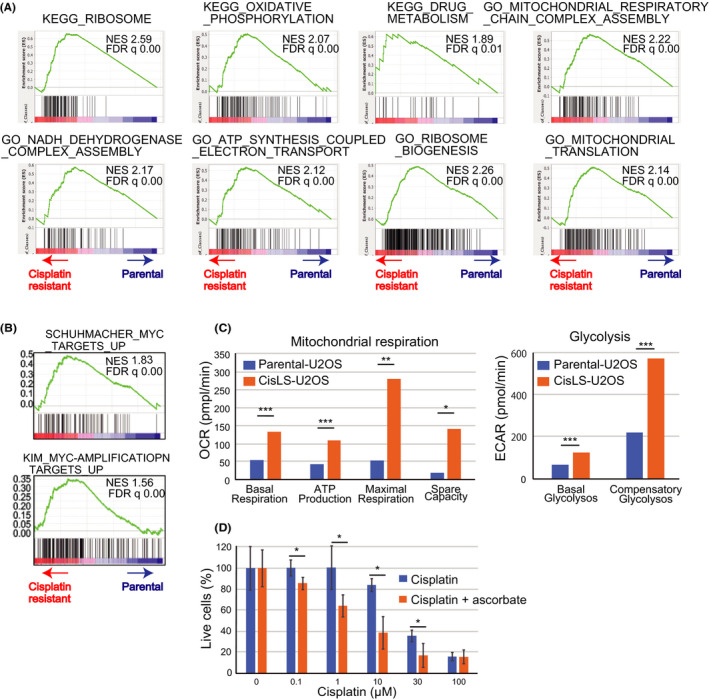
Ascorbate treatment sensitizes cisplatin‐resistant U2OS cells to cisplatin treatment. A, RNA sequence analysis revealed that ribosome biogenesis and mitochondrial respiration were enhanced in CisLS‐U2OS cells. B, Upregulation of Myc target genes in CisLS‐U2OS cells. C, Mitochondrial (basal respiration, ATP production, maximal respiration, and spare capacity) (left) and glycolytic functions (right) were measured by a flux analyzer in parental and CisLS‐U2OS cells (n = 3). D, Parental and CisLS‐U2OS cells (1x10^4^ cells) were treated with cisplatin (0‐100 µmol/L) or cisplatin plus ascorbate (10 µmol/L) for 96 h and the number of live cells were measured by cell proliferation assay (n = 6). Data are presented as the mean ± SD from three independent experiments. *P < .05; **P < .01; ***P < .001

Acquired chemoresistance of cancer cells is both a huge concern and a limiting factor for chemotherapy programs. Our next objective, therefore, was to establish whether ascorbate treatment restores the cisplatin sensitivity of cisplatin‐resistant cells. We first established U2OS cell lines that were less sensitive to cisplatin (CisLS‐U2OS) by continuous exposure of parental U2OS cells to increasing doses of cisplatin over 6 months. The IC_50_ value of 12.4 µmol/L in parental U2OS cells was shifted to 21.4 µmol/L in CisLS‐U2OS cells (Figure S1). To characterize the CisLS‐U2OS cells, we analyzed the gene expression by RNA sequencing (Figure S2A).^41^ A GSEA analysis was performed to identify differentially expressed gene profiles on comparison of the CisLS‐U2OS cell line to parental cell lines. GSEA revealed that genes associated with ribosome biogenesis, mitochondrial function (including oxidative phosphorylation and respiratory chain function), and drug metabolism were enriched in CisLS‐U2OS cells (Figure 6A and Figure S2B,C). Recent evidence indicates that MYC stimulates ribosomal biogenesis. Hence, using GSEA, we analyzed whether MYC target genes are induced in CisLS‐U2OS cells, using two data sets for MYC target genes.^42^, ^43^ MYC target genes were indeed enriched in CisLS‐U2OS cells (Figure 6B).

To further characterize the effects of cisplatin resistance, we assessed glycolysis and mitochondrial function in CisLS‐U2OS cells relative to parental cells. Parameters of mitochondrial respiration, such as basal respiration, ATP synthesis, maximal respiration levels, and spare respiration, were markedly increased in CisLS‐U2OS cells (Figure 6C, Left), which is consistent with the presence of genes for mitochondrial respiration in the GSEA. The additional observation of enhanced glycolysis in CisLS‐U2OS cells suggests that the metabolic reprogramming of both the glycolytic pathway and mitochondrial function is an important process in the development of cisplatin resistance (Figure 6C, Right).

Finally, to test whether ascorbate restores cisplatin sensitivity in CisLS‐U2OS cells, we examined the cellular viability of parental and CisLS‐U2OS cells treated with cisplatin or cisplatin plus ascorbate. The addition of ascorbate shifted the IC_50_ value of 21.6 µmol/L (for cisplatin treatment of CisLS‐U2OS cells) to 3.61 µmol/L (Figure 6D). Thus, ascorbate overcomes cisplatin resistance.

## 4 DISCUSSION

Chemoresistance is a major cause of cancer mortality. The response to preoperative chemotherapy is a particularly important predictive factor for the prognosis of OS patients.^7^ Although the mechanisms underlying chemoresistance are complex and may vary among cancers, increasing evidence supports that heterogeneity is a driving force for chemoresistance in general,^44^ and that the existing cell populations with chemoresistance can confer intrinsic resistance to chemotherapy. Supporting this view, cisplatin‐resistant OS cells possess stem‐like properties,^45^ a small population of such CSCs having been recognized as drivers for tumor resistance and recurrence in a wide range of cancers.

In the present study, cisplatin treatment induced robust induction of γH2AX, which correlated with raised intracellular ROS levels. Ascorbate enhanced cisplatin‐induced suppression of cell viability and proliferation by further increasing ROS production, and causing enhanced DNA damage, mitochondrial dysfunction, and metabolic shift. Cisplatin‐resistant U2OS cells exhibited increased ribosome biogenesis, ATP synthesis, and mitochondrial function, as might be anticipated from the important effects of cisplatin on oxidative stress. Ascorbate not only enhances the effect of cisplatin on OS cell lines, caused by oxidative stress, but sensitizes cisplatin‐resistant U2OS cells to cisplatin‐induced cytotoxicity.

The mechanisms underlying mitochondrial impairment and subsequent ROS production induced by cisplatin treatment are fully understood. Both involvement of mtDNA^46^, ^47^ and metabolic reprogramming have been proposed, in addition to conventional DNA damage. One proposal is that cisplatin enhances ROS production as a consequence of its direct effect on mtDNA, leading to impairment of the electron transport chain. Ascorbate has also been reported to increase ROS production in some cancer cells.^48^ The resulting high‐dose ROS induces irreversible oxidative damage of DNA, proteins, and lipids ^49^ and interferes with critical cellular functions.^50^ Consistent with such reports, our combined treatment of U2OS cells with cisplatin and ascorbate induced higher production of ROS (relative to cisplatin alone) and suppressed mitochondrial functions.

The effect of ROS in cells is complex. Certain ROS act in signaling pathways as part of the normal physiology of the cell. However, the cellular redox state is in a delicate balance and its homeostasis is crucial, attained by precise regulation of ROS formation and removal, with the help of scavenging enzymes and antioxidant agents. Disruption of this balance is a feature of many diseases, including cancer. In many cancer cells, intracellular ROS levels are higher than in normal cells due to alterations in the microenvironment, activation of oncogenes, and dysregulation of metabolism.^51^, ^52^, ^53^ Indeed, a moderate increase in ROS, which promotes cell proliferation and genetic mutations, is considered a driving force of tumorigenesis. ROS removal pathways are constitutively active^54^ in cancer cells as they attempt to circumvent ROS damage and maintain the levels lower than the toxic threshold. This constitutive response to the high oxidative stress levels in cancer cells actually confers greater vulnerability on them to a surge of intracellular ROS induced by exogenous agents, such as cisplatin and ascorbate.

Here, we have observed a clear synergistic effect of ascorbate on the effectiveness of cisplatin and have demonstrated increased ROS production. It is highly possible that ascorbate exerts its effect simply by further increasing oxidative stress, as inferred above. However, other possible modes of action for ascorbate should be considered. For example, ascorbate regulates the expression of the ten‐eleven translocation (TET) enzymes and hypoxia‐inducible transcription factors (HIFs). By acting as a co‐factor for TET enzymes, ascorbate facilitates DNA demethylation.^55^ Thus, ascorbate treatment may reactivate tumor suppressor genes silenced by DNA methylation in cancer cells, causing increased susceptibility to chemotherapeutic agents in this way. TET‐mediated antitumor activity has been confirmed in myelodysplastic syndrome.^56^, ^57^ Ascorbate is also critical for the function of HIFs. HIF protein stability is negatively regulated by hydroxylation of HIF‐1α subunits. Under hypoxic conditions, HIF‐1 shifts metabolic states from aerobic to anaerobic metabolism by increasing flux through glycolysis. Since ascorbate is required for hydroxylation, ascorbate treatment decreases HIF‐1 activity.^23^, ^58^ Supporting this, tumor growth was inhibited by ascorbate in vivo, with decreased HIF‐1 activity and therefore, decreased glycolytic flux.^59^ Indeed, there is considerable amount of literature on describing a synergistic effect of ascorbate on other chemotherapeutic agents in vivo and different mechanisms for these effects are possible.^60^, ^61^ Here, a high proportion of our data, including consistent enhancement of metabolic changes induced by cisplatin‐induced oxidative stress, support a synergistic role for ascorbate on cisplatin treatment of OS that is mediated via enhanced ROS production.

CSCs are thought to be responsible for the initiation, drug resistance, and recurrence of tumors.^62^, ^63^ Thus, any strategies to efficiently eradicate CSCs will increase treatment efficacy. CSCs have a unique mechanism for maintaining ROS at low levels, which protects them from DNA damage and cell death induced by ROS production.^64^, ^65^, ^66^ In our studies, U2OS sphere formation was markedly reduced upon combined treatment with cisplatin and ascorbate, the reasons for which are unclear. Notably, high mitochondrial mass is a common and characteristic feature of CSCs.^67^, ^68^, ^69^ The marked impact of the combined treatment on sphere formation is presumably linked to the significant mitochondrial activity in the cell populations with CSC properties, in which cisplatin and ascorbate acting together have a sufficient deleterious effect. Our findings are indicative of combination therapy decreasing CSC survival, but further experiments are required to precisely address this issue. An improved effect of combination therapy on CSCs in OS would be a major step forward, given that the success of preoperative chemotherapy is a key prognostic indicator.

Combined treatment with cisplatin and ascorbate increased intracellular ROS and depressed energy efficiency. Consistent with our flux analyzer data, the expression of genes enhancing glycolytic flux was upregulated, including hexokinase 2 (*HK2*),^70^ *MCT4*, and *G6PD*. The PPP is often activated in cancer cells and enhances the supply of ribose, membrane synthesis, and antioxidants.^71^, ^72^ Metabolic intermediates and downstream products of PPP, such as glutathione, confer resistance to chemotherapy. G6PD is a rate‐limiting enzyme of the PPP. The increase in expression of all four enzymes treated with cisplatin plus ascorbate supports the induction of glycolysis flux, presumably as a self‐defense mechanism in U2OS cells upon greater disruption to mitochondrial function. Cisplatin treatment thus alters metabolism, which has strong implications for improvements to cisplatin responsiveness and suggests that a metabolomics analysis could be of predictive value for determining likely cisplatin sensitivity in OS patients.

The ribosome biogenesis pathway is the most enriched gene signature in CisLS‐U2OS cells. Under environmental stress, ribosomes function to organize the expression of stress‐response genes.^73^ The signaling pathways activated or inactivated in cisplatin‐resistant cells are not fully understood, but a link between cisplatin and ribosome biogenesis has been observed in multiple cancer cell lines.^74^ For example, ribosomal protein L36 contributes to establishing cisplatin resistance in human cancer cells.^75^ Expression levels of ribosomal protein L37 affect cell cycle arrest and DNA damage response after cisplatin exposure.^76^ These reports, together with our data, indicate an intimate mechanistic link between cisplatin resistance and the ribosomal stress response. Although further studies are necessary, our RNA sequence data suggest that the modification of ribosome biogenesis is dominant in cisplatin resistance in U2OS cells, which is at least partly due to MYC activation. In support of our findings, neuroblastoma with N‐Myc overexpression become resistant to cisplatin^77^ and cisplatin exposure activates c‐Myc in head and neck squamous carcinoma.^78^

In summary, the current study has identified a convergence of phenotypic and detailed metabolic transitions during short‐ and long‐term cisplatin treatment. We propose that combined treatment with cisplatin and ascorbate efficiently suppresses the proliferation and sphere formation of human OS cells via ROS production and a significant metabolic shift. Importantly, the effects of a combination of cisplatin and ascorbate extend to sphere formation and, therefore, the stem‐like cells in OS, which are often drug‐resistant. Effective suppression of CSC growth, in addition to other tumor cells, could improve early treatment and survival in OS patients. In addition, potential therapeutic targets to circumvent cisplatin resistance have been identified by RNA sequencing analysis, which provides a potential platform for testing novel combinatorial therapies. We anticipate that these approaches will provide new therapeutic options for managing OS patients in the future.

## CONFLICT OF INTEREST

The authors declare that there is no conflict of interest.

## AUTHOR CONTRIBUTIONS

Participated in research design: Oka, Nishimura, Akagi, and Okada. Conducted experiments: Oka, Komuro, Amano, Dash, Ota, Honda, Ueda, and Okada. Contributed new reagents or analytic tools: Ueda. Performed data analysis: Oka, Ueda, and Okada. Wrote or contributed to the writing of the manuscript: Oka, Ueda, Akagi, and Okada.

## Supporting information

Fig S1‐S2

## Data Availability

The RNA sequence data have been deposited in the DDBJ database (accession number: DRA009407).
