# Supplementary material for: Ascorbate sensitizes human osteosarcoma cells to the cytostatic effects of cisplatin
Source: Pharmacol Res Perspect. 2020 Jul 29;8(4):e00632. doi: 10.1002/prp2.632 (PMC7387887; doi:10.1002/prp2.632)
Supplement: Supplementary file 1 — Fig S1‐S2 [file PRP2-8-e00632-s001.pdf]

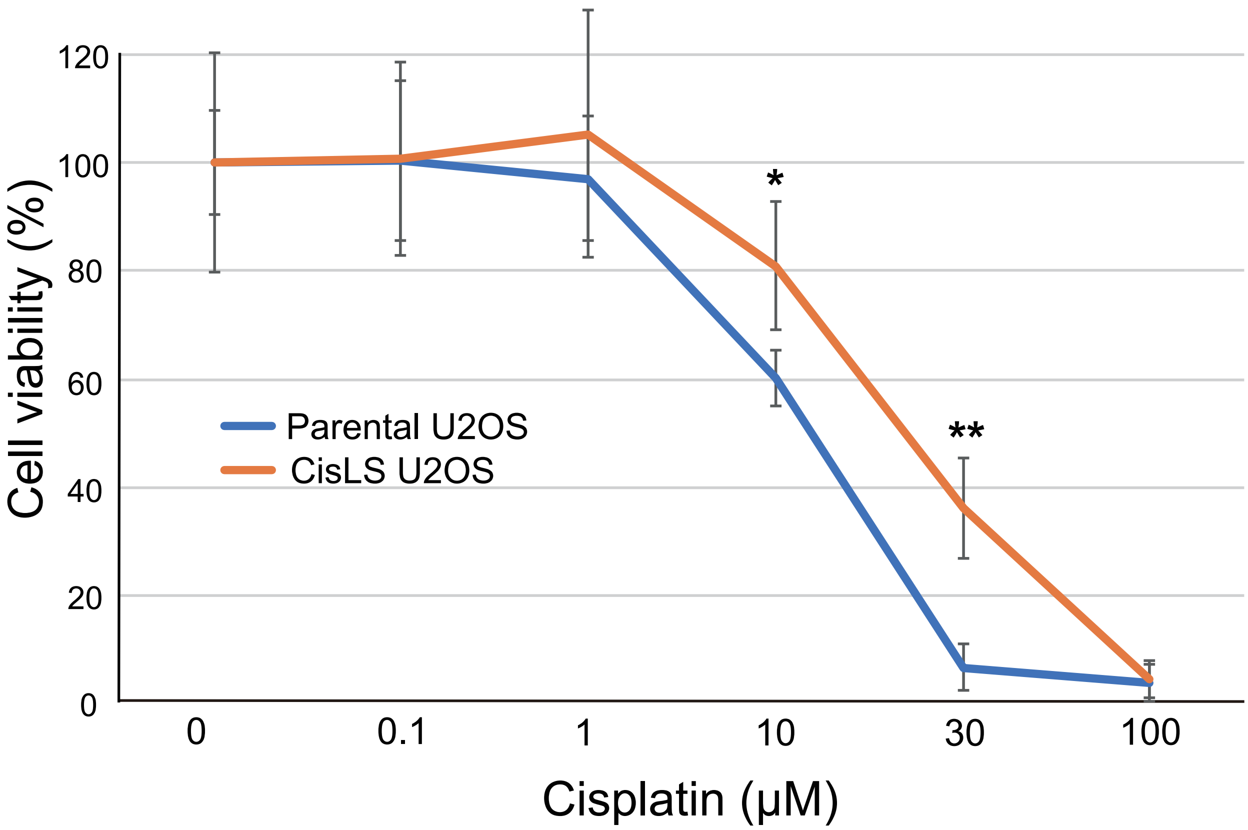

Fig. S1. Dose response curves for parental and cisplatin-resistant U2OS cells.

The cell viability assay was performed. The IC50 value for cisplatin was calculated from the curves constructed by plotting cellular viability versus drug concentration. Data are presented as the mean ± SD (n=6) from three independent experiments. *$p < 0.05$; **$p < 0.01$.

Oka et al Fig.S2

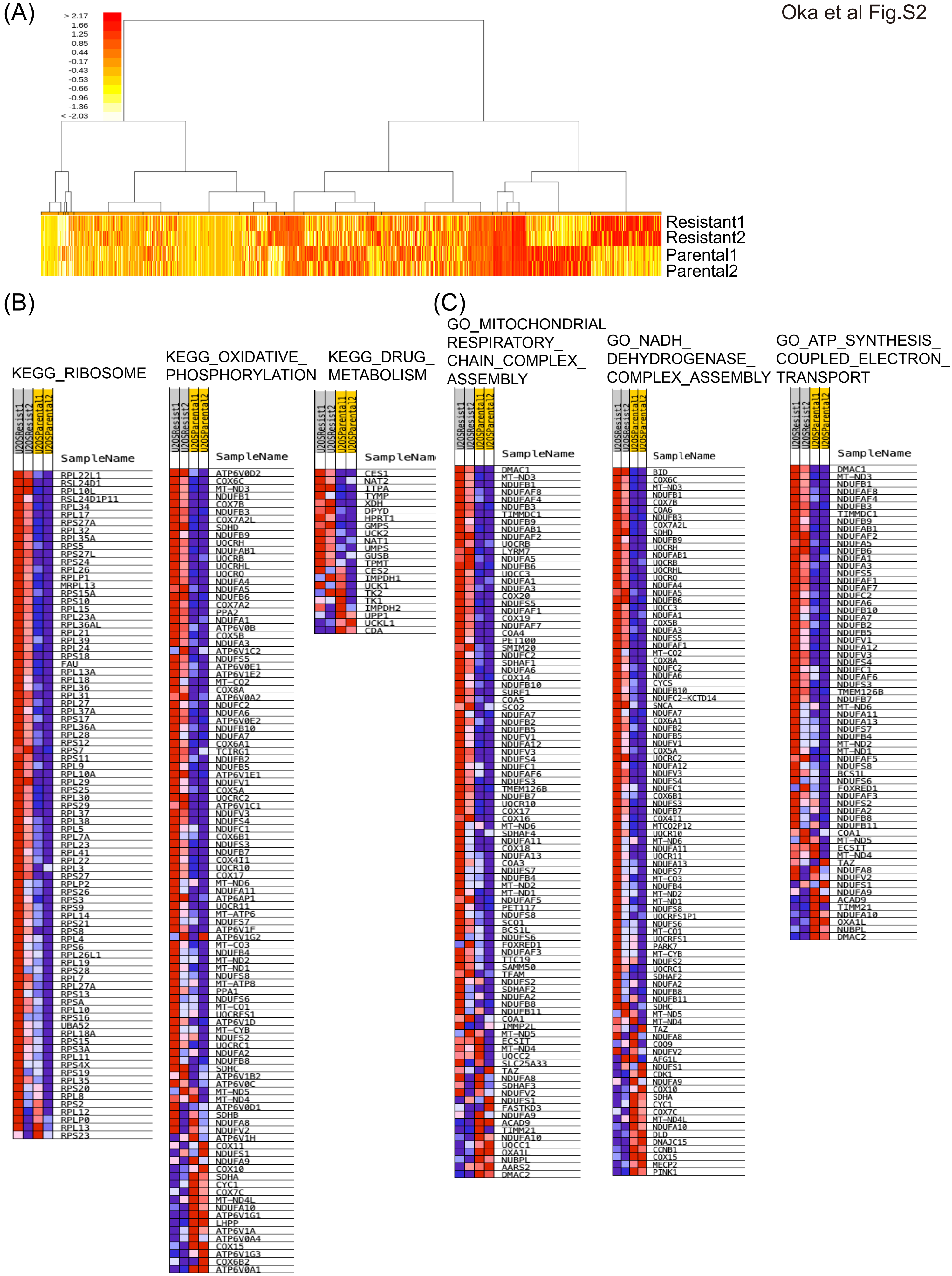

Fig. S2. Distinct gene expression signatures of parental and cisplatin-resistant U2OS cell lines.
(A) Heat map and clustering analysis of RNA sequence data from two parental and cisplatin U2OS cell lines. The data were analyzed by TCC software.(Sun et al., 2013) (B) Enriched upregulated gene sets in cisplatin U2OS cell lines obtained by GSEA.
